# Supplementary material for: Mycobiota of Mexican Maize Landraces with Auxin-Producing Yeasts That Improve Plant Growth and Root Development
Source: Plants (Basel). 2023 Mar 15;12(6):1328. doi: 10.3390/plants12061328 (PMC10058334; doi:10.3390/plants12061328)
Supplement: Supplementary file 1 [file plants-12-01328-s001.zip › plants-2238055-supplementary.pdf]

Supplementary table S1. Yeast strains isolated from red and blue corn plants and their plant growth-promoting characteristics‡

Strain	GenBank ID	Maize variety	Plant Sample	Molecular identification	Siderophore production[1] (% siderophore units)	Auxin Production[2] (µg/mL)	Phosphate Solubilization[3] (solubilization index)	Pectinase production[4] (enzymatic index)	Cellulase production[5] (enzymatic index)	Protease production[6] (enzymatic index)	Amylase production[7] (enzymatic index)
Ry1	MN299236	Red	Cob	*Rhodotorula mucilaginosa*	ND	ND	2.0±0c	ND	ND	ND	ND
Ry2	MN299256	Red	Cob	*Clavispora lusitaniae*	ND	ND	2.0±0c	ND	ND	ND	ND
Ry3	MN299237	Red	Cob	*Rhodotorula mucilaginosa*	ND	ND	2.0±0c	ND	ND	ND	ND
Ry4	MN299230	Red	Cob	*Suhomyces prunicola*	ND	ND	3.0±0d	ND	ND	ND	ND
Ry5	MN299257	Red	Cob	*Clavispora lusitaniae*	ND	ND	ND	ND	ND	ND	ND
Ry6-2	MN299222	Red	Cob	*Candida oleophila/Candida railenensis*	ND	ND	1.5±0b	ND	ND	ND	ND
Ry7	MN299234	Red	Cob	*Rhodotorula mucilaginosa*	ND	ND	ND	ND	ND	ND	ND
Ry8	MN299235	Red	Cob	*Rhodotorula mucilaginosa*	ND	ND	ND	ND	ND	ND	ND
Ry9	MN299258	Red	Phyllosphere	*Clavispora lusitaniae*	ND	ND	ND	ND	ND	ND	ND
Ry11	MN299228	Red	Phyllosphere	*Kurtzmaniella quercitrusa*	ND	ND	2.1±0c	ND	ND	ND	ND
Ry13	MN299259	Red	Phyllosphere	*Clavispora lusitaniae*	ND	ND	1.1±0a	ND	ND	ND	ND
Ry14	MN299260	Red	Phyllosphere	*Clavispora lusitaniae*	ND	ND	1.1±0a	ND	ND	ND	ND
Ry14-2	MN299261	Red	Phyllosphere	*Clavispora lusitaniae*	ND	ND	ND	ND	ND	ND	ND
Ry15	MN299262	Red	Phyllosphere	*Clavispora lusitaniae*	ND	ND	ND	ND	ND	ND	ND
Ry16	MN299263	Red	Phyllosphere	*Clavispora lusitaniae*	ND	ND	ND	ND	ND	ND	ND

Ry17	MN299264	Red	Phyllosphere	*Clavispora lusitaniae*	ND	ND	ND	ND	ND	ND	ND
Ry18	MN299265	Red	Phyllosphere	*Clavispora lusitaniae*	ND	ND	ND	ND	ND	ND	ND
Ry19	MN299229	Red	Phyllosphere	*Holtermanniella takashimae*	ND	ND	ND	ND	ND	ND	ND
Ry20	MN299266	Red	Phyllosphere	*Clavispora lusitaniae*	ND	ND	ND	ND	ND	ND	ND
Ry21	MN299267	Red	Phyllosphere	*Clavispora lusitaniae*	ND	ND	ND	ND	ND	ND	ND
Ry22	MN299268	Red	Leaf endosphere	*Clavispora lusitaniae*	ND	ND	ND	ND	ND	ND	ND
Ry23	MN299238	Red	Root endosphere	*Rhodotorula mucilaginosa*	ND	ND	ND	ND	ND	ND	ND
Ry24	MN299221	Red	Root endosphere	*Candida oleophila/Candida railenensis*	ND	ND	1.2±0a	ND	ND	ND	ND
Ry25	MN299233	Red	Root endosphere	*Rhodotorula mucilaginosa*	ND	ND	ND	ND	ND	ND	ND
Ry26	MN299223	Red	Root endosphere	*Candida oleophila/ Candida railenensis*	ND	ND	1.2±0a	ND	1.1±0	ND	ND
Ry27	MN299239	Red	Root endosphere	*Rhodotorula mucilaginosa*	ND	ND	1.5±0b	ND	ND	ND	ND
Ry29	MN299255	Red	Root endosphere	*Solicoccozyma* sp.	ND	ND	ND	ND	ND	ND	ND
Ry30	MN299269	Red	Root endosphere	*Clavispora lusitaniae*	ND	ND	ND	1.1±0a	ND	ND	ND

Ry31	MN299254	Red	Root endosphere	*Solicoccozyma* sp.	ND	9.3±2.4b	ND	ND	ND	ND	ND
Ry32	MN299289	Red	Root endosphere	*Papiliotrema flavescens*	ND	ND	ND	ND	1.1±0	ND	ND
Ry33	MN299232	Red	Root endosphere	*Rhodotorula mucilaginosa*	ND	ND	ND	ND	ND	ND	ND
Ry34	MN299240	Red	Root endosphere	*Rhodotorula mucilaginosa*	ND	ND	ND	ND	ND	ND	ND
Ry35	MN299241	Red	Root endosphere	*Rhodotorula mucilaginosa*	ND	ND	ND	ND	ND	ND	ND
Ry37	MN299242	Red	Root endosphere	*Rhodotorula mucilaginosa*	ND	ND	ND	ND	ND	ND	ND
Ry39	MN299243	Red	Root endosphere	*Rhodotorula mucilaginosa*	ND	ND	ND	ND	ND	ND	ND
Ry40	MN299270	Red	Root endosphere	*Clavispora lusitaniae*	ND	ND	ND	ND	ND	ND	ND
Ry41	MN299231	Red	Cob	*Suhomyces prunicola*	ND	ND	ND	ND	ND	ND	ND
Y2	MN299290	Blue	Phyllosphere	*Papiliotrema flavescens*	ND	ND	1.7±0b	ND	ND	ND	ND
Y3	MN299271	Blue	Phyllosphere	*Clavispora lusitaniae*	ND	ND	ND	ND	ND	ND	ND
Y4	MN299272	Blue	Phyllosphere	*Clavispora lusitaniae*	ND	ND	ND	ND	ND	ND	ND
Y5	MN299298	Blue	Phyllosphere	*Papiliotrema flavescens*	ND	ND	ND	ND	ND	ND	ND
Y7	MN299273	Blue	Phyllosphere	*Clavispora lusitaniae*	ND	ND	ND	ND	ND	ND	ND

Y9	MN299274	Blue	Phyllosphere	*Clavispora lusitaniae*	ND	ND	ND	ND	ND	ND	ND
Y10	MN299275	Blue	Phyllosphere	*Clavispora lusitaniae*	ND	ND	ND	ND	ND	ND	ND
Y11	MN299292	Blue	Phyllosphere	*Clavispora lusitaniae*	ND	4±0.3d	ND	ND	ND	ND	ND
Y12	MN299301	Blue	Phyllosphere	*Saitozyma paraflava*	ND	ND	ND	ND	ND	ND	ND
Y14	MN299276	Blue	Phyllosphere	*Clavispora lusitaniae*	ND	ND	ND	ND	ND	ND	ND
Y16	MN299224	Blue	Phyllosphere	*Candida oleophila/Candida railenensis*	ND	ND	ND	ND	ND	ND	ND
Y17	MN299225	Blue	Phyllosphere	*Candida oleophila/Candida railenensis*	ND	ND	ND	ND	ND	ND	ND
Y19	MN299302	Blue	Phyllosphere	*Naganishia* sp.	ND	ND	ND	4.0±0d	ND	ND	ND
Y20	MN299293	Blue	Phyllosphere	*Candida oleophila/ Candida railenensis*	ND	ND	ND	ND	ND	ND	ND
Y21	MN299294	Blue	Phyllosphere	*Papiliotrema flavescens*	ND	ND	ND	ND	ND	ND	ND
Y22	MN299277	Blue	Phyllosphere	*Clavispora lusitaniae*	ND	ND	ND	ND	ND	ND	ND
Y23	MN299247	Blue	Phyllosphere	*Rhodotorula glutinis*	ND	7.1±0.1c	ND	ND	ND	ND	ND
Y24	MN299244	Blue	Phyllosphere	*Rhodotorula mucilaginosa*	ND	ND	ND	ND	ND	ND	ND
Y27	MN299295	Blue	Phyllosphere	*Papiliotrema flavescens*	87±4 a	ND	ND	ND	ND	ND	ND
Y30	MN299226	Blue	Phyllosphere	*Candida oleophila/Candida railenensis*	ND	ND	ND	ND	ND	ND	ND
Y31	MN299278	Blue	Phyllosphere	*Clavispora lusitaniae*	ND	ND	1.0±0a	ND	ND	ND	ND
Y32	MN299296	Blue	Phyllosphere	*Papiliotrema flavescens*	ND	ND	ND	ND	ND	ND	ND

Y33	MN299279	Blue	Phyllosphere	*Clavispora lusitaniae*	ND	ND	ND	ND	ND	ND	ND
Y35	MN299245	Blue	Leaf endosphere	*Rhodotorula mucilaginosa*	ND	ND	ND	ND	ND	ND	ND
Y40	MN299227	Blue	Leaf endosphere	*Candida oleophila/Candida railenensis*	ND	ND	ND	ND	ND	ND	ND
Y41	MN299248	Blue	Leaf endosphere	*Rhodotorula glutinis*	57±3 b	ND	ND	ND	1.5±0a	3.0±0c	ND
Y42	MN299297	Blue	Leaf endosphere	*Papiliotrema flavescens*	ND	ND	1.0±0a	ND	ND	ND	ND
Y43	MN299249	Blue	Leaf endosphere	*Rhodotorula glutinis*	ND	ND	ND	ND	1.7±0a	1.6±0a	ND
Y44	MN299250	Blue	Leaf endosphere	*Rhodotorula glutinis*	ND	ND	ND	ND	1.6±0a	ND	ND
Y46	MN299246	Blue	Leaf endosphere	*Rhodotorula mucilaginosa*	ND	ND	ND	ND	ND	ND	ND
Y47	MN299280	Blue	Leaf endosphere	*Clavispora lusitaniae*	ND	ND	ND	ND	ND	ND	ND
Y48	MN299298	Blue	Leaf endosphere	*Papiliotrema flavescens*	ND	ND	ND	ND	ND	ND	ND
Y50	MN299251	Blue	Leaf endosphere	*Rhodotorula glutinis*	ND	ND	ND	ND	ND	ND	ND
Y52	MN299303	Blue	Leaf endosphere	*Naganishia* sp.	ND	15.8±1.8a	ND	1.5±0b	ND	ND	ND

Y53	MN299304	Blue	Leaf endosphere	*Naganishia* sp.	ND	ND	ND	1.25±0a	ND	ND	ND
Y55	MN299281	Blue	Leaf endosphere	*Clavispora lusitaniae*	ND	ND	ND	ND	ND	ND	ND
Y57	MN299252	Blue	Leaf endosphere	*Rhodotorula glutinis*	ND	ND	ND	ND	ND	2.4±0b	ND
Y58	MN299253	Blue	Leaf endosphere	*Rhodotorula glutinis*	ND	ND	ND	ND	2.6±0b	1.6±0a	ND
Y59	MN299282	Blue	Leaf endosphere	*Clavispora lusitaniae*	ND	ND	ND	ND	ND	ND	ND
Y63	MN299305	Blue	Leaf endosphere	*Aureobasidium pullulans*	ND	ND	ND	ND	ND	ND	ND
Y64	MN299306	Blue	Leaf endosphere	*Aureobasidium pullulans*	ND	ND	ND	1.8±0c	3.4±0c	2.6±0b	ND
Y65	MN299299	Blue	Leaf endosphere	*Papiliotrema flavescens*	ND	ND	ND	ND	1.0±0a	ND	ND
Y66	MN299283	Blue	Leaf endosphere	*Clavispora lusitaniae*	ND	ND	ND	ND	ND	ND	ND
Y68	MN299284	Blue	Leaf endosphere	*Clavispora lusitaniae*	ND	ND	ND	ND	ND	ND	ND
Y69	MN299285	Blue	Phyllosphere	*Clavispora lusitaniae*	ND	ND	ND	ND	ND	ND	ND
Y70	MN299300	Blue	Phyllosphere	*Papiliotrema flavescens*	ND	ND	ND	ND	ND	ND	ND

Y71	MN299307	Blue	Phyllosphere	*Papiliotrema flavescens*	ND	ND	ND	ND	ND	ND	ND
Y74	MN299286	Blue	Phyllosphere	*Clavispora lusitaniae*	ND	ND	ND	ND	ND	ND	ND
Y75	MN299287	Blue	Phyllosphere	*Clavispora lusitaniae*	ND	ND	ND	ND	ND	ND	ND
Y76	MN299288	Blue	Phyllosphere	*Clavispora lusitaniae*	ND	ND	ND	ND	ND	ND	ND

‡ The mean ± standard deviation of 3 experiments is shown. Different lower-case letters between strains indicate statistically significant differences (p-value <0.05) according to ANOVA with Tukey post hoc. ND= Not detected.

[1] Siderophore production was detected in Grimm-Allen cultures and quantified by CAS-Fe solution.

[2] Auxin production was detected in YPD-L-tryptophan cultures and quantified with Salkowski reagent.

[3] Phosphate solubilization was evaluated using Pikovskaya medium with tricalcium phosphate.

[4] Pectinase production was evaluated in minimal medium with pectin and revealed using a 5% CTAB solution.

[5] Cellulase production was evaluated using Congo red medium.

[6] Protease production was evaluated using Skim milk medium.

[7] Amylase production was evaluated in minimal medium with potato starch and revealed using an Iodine solution.
